# A review on the role of LINC01133 in cancers

**DOI:** 10.1186/s12935-022-02690-z

**Published:** 2022-08-30

**Authors:** Soudeh Ghafouri-Fard, Tayyebeh Khoshbakht, Bashdar Mahmud Hussen, Mohammad Taheri, Majid Mokhtari

**Affiliations:** 1 Department of Medical Genetics, School of Medicine, Shahid Beheshti University of Medical Sciences, Tehran, Iran; 2 Phytochemistry Research Center, Shahid Beheshti University of Medical Sciences, Tehran, Iran; 3 Department of Pharmacognosy, College of Pharmacy, Hawler Medical University, Kurdistan Region, Erbil, Iraq; 4 Institute of Human Genetics, Jena University Hospital, Jena, Germany; 5 Urology and Nephrology Research Center, Shahid Beheshti University of Medical Sciences, Tehran, Iran; 6 Skull Base Research Center, Loghman Hakim Hospital, Shahid Beheshti University of Medical Sciences, Tehran, Iran

**Keywords:** LINC01133, cancer, Biomarker

## Abstract

Long Intergenic Non-Protein Coding RNA 1133 (LINC01133) is a long non-coding RNA (lncRNA) which interacts with miR-106a-3p, miR-576-5p, miR-495-3p, miR-205, miR-199a-5p, miR-4784, miR-30a-5p, miR-199a, miR-30b-5p, miR-216a -5p and miR-422a, thus increasing expression of mRNA targets of these miRNAs. LINC01133 can affect cancer metastasis through regulation of epithelial-mesenchymal transition program. Dysregulation of this lncRNA has been repeatedly detected in the process of tumorigenesis. In this review, we summarize the results of various studies that reported dysregulation of LINC01133 in different samples and described the role of this lncRNA as a marker for these disorders.

## Introduction

Long non-coding RNAs (lncRNAs) have been vastly investigated for their effects in the carcinogenesis [1]. These transcripts have sizes larger than 200 nt and are mainly located in the nucleus [2]. Although lncRNAs are expressed at low levels, they participate in transcriptional and post-transcriptional regulation of gene expression via interacting with other types of biomolecules, namely nucleic acids or proteins [3]. They can enhance or interfere with establishment of transcription loops. Moreover, they are able to induce or suppress recruitment of other regulators [4, 5] and affect mRNA splicing. Finally, they serve as origin for microRNAs (miRNAs) [6]. Notably, lncRNAs can affect tumorigenesis through acting as oncogenes or tumor suppressors [7].

Long Intergenic Non-Protein Coding RNAs (LINC RNAs), as a class of lncRNAs have been found to interplay with chromatin modification complexes or RNA binding proteins [8]. These transcripts can change gene expression programs. Previous studies have reported distinctive expression profile of LINC RNAs in primary and metastatic tumors [8, 9] and the role of these transcripts in the metastases [10–12]. Moreover, expression of these transcripts is finely controlled in the course of development and in response to different signals [13]. LINC01133 is an example of these transcripts. The gene coding this lncRNA is located on 1q23.2. This lncRNA has four transcript variants with sizes of 1996 bp, 1418 bp, 1405 bp and 1266 bp, respectively (http://www.ensembl.org/Homo_sapiens/Gene/Summary?db=core;g=ENSG00000224259;r=1:159958035-159984750).

LINC01133 has been found to be dysregulated in the process of tumorigenesis. However, it has different patterns of expression in various malignancies, or even within a certain type of malignancy. In this review, we summarize the results of various studies that reported dysregulation of LINC01133 in cell line originated from different cancer types, animal studies and investigations in human samples.

## Cell line studies

**Fig. 1:**
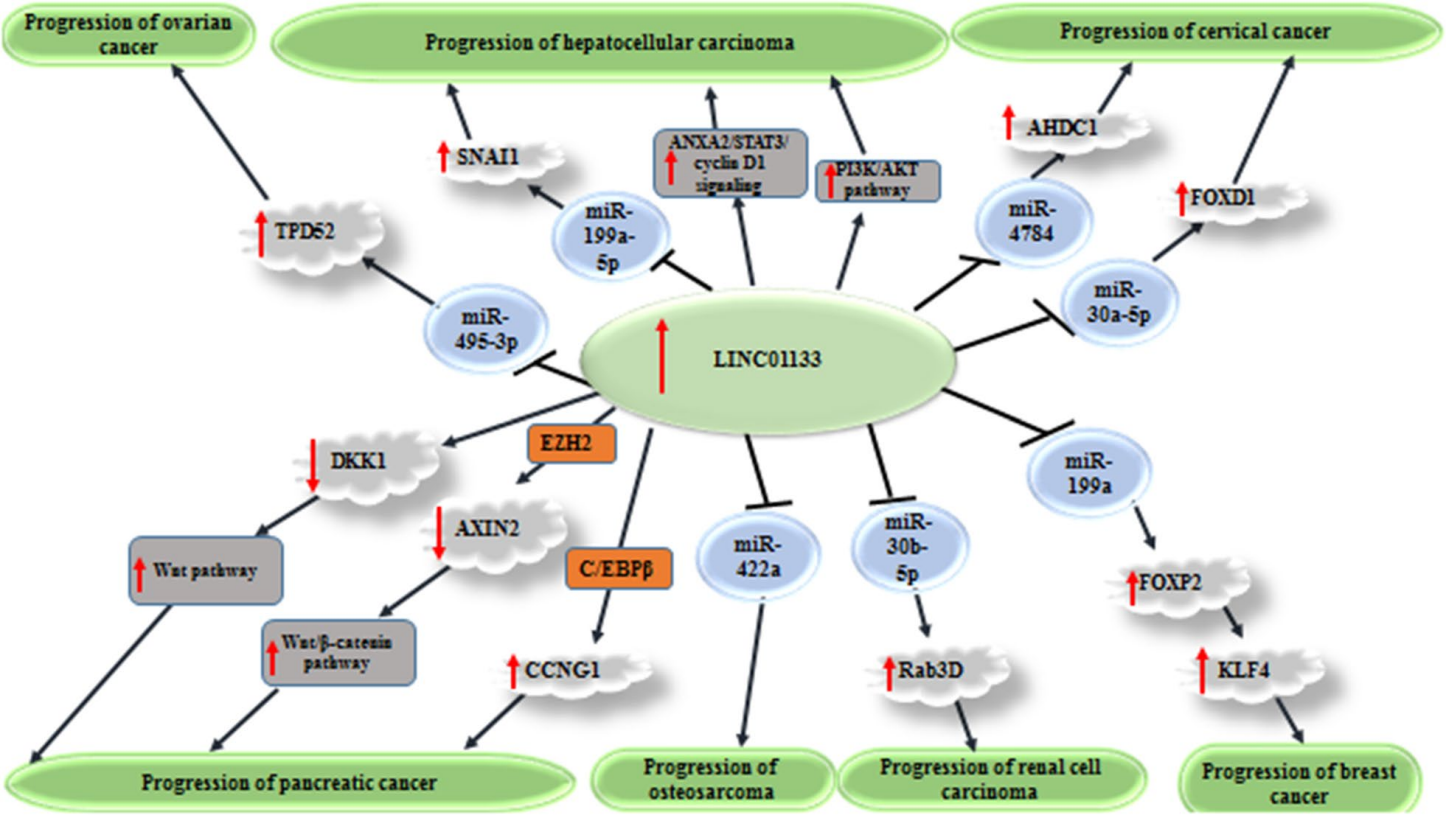
Oncogenic roles of LINC01133 in cancers. Detailed information about mechanism of involvement of LINC01133 in these cancers is provided in Table 1. ↑ shows up-regulation. ┴ shows inhibitory effect

**Fig. 2:**
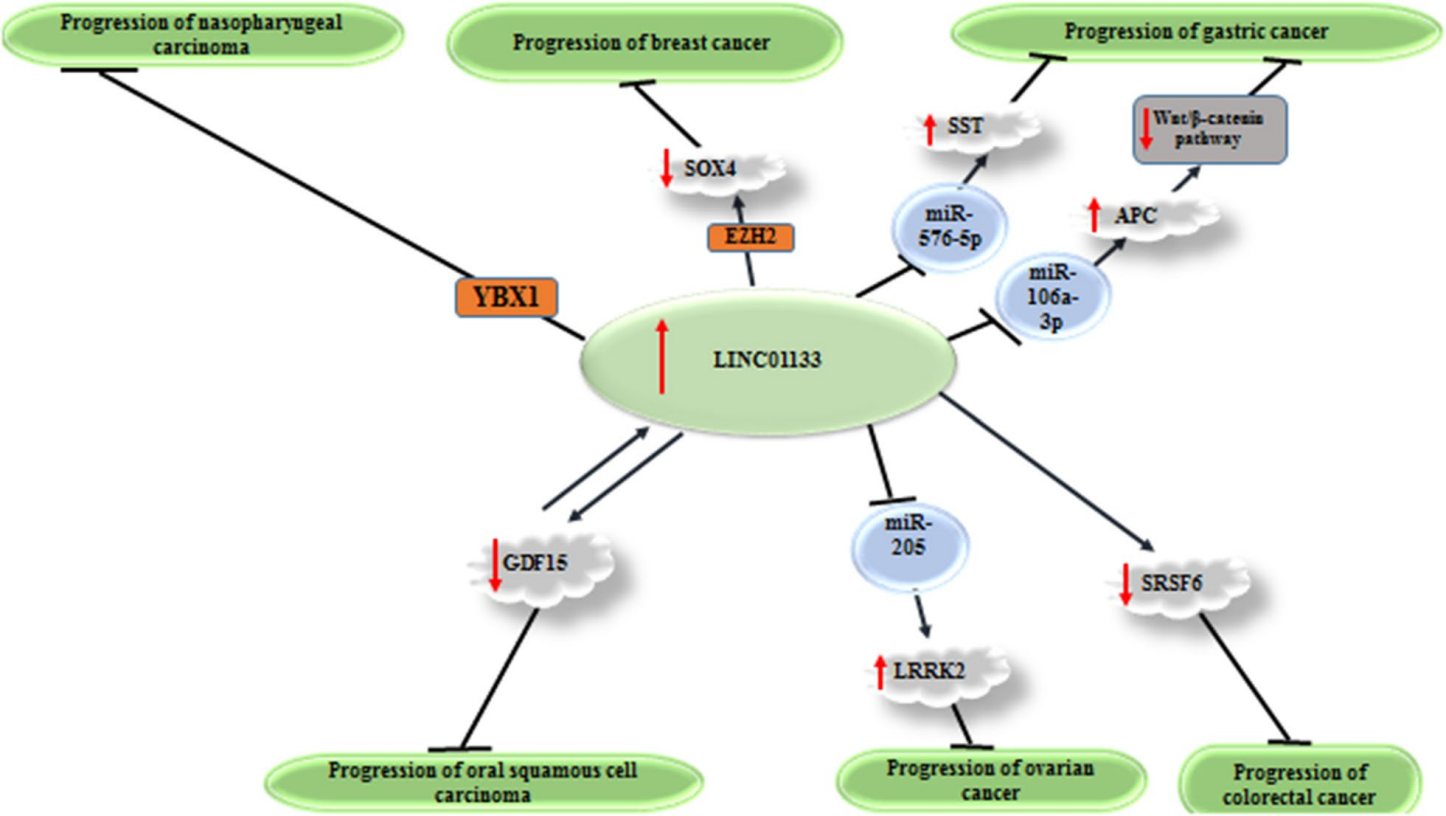
Tumor suppressor roles of LINC01133 in cancers. Detailed information about mechanism of involvement of LINC01133 in these cancers is provided in Table 1. ↑ shows up-regulation.┴ shows inhibitory effect

In vitro and functional studies in different cell lines have reported either oncogenic (Fig. 1) or tumor suppressor role (Fig. 2) for LINC01133. In the following sections, we describe the role of LINC01133 in different cancers.

## Gynecological cancers

Expression of LINC01133 has been found to be enhanced in epithelial ovarian cancer cell lines. Functionally, LINC01133 enhances migration and invasiveness of ovarian cancer cells. LINC01133 and miR-495-3p have been shown to reciprocally repress expression of each other. LINC01133 can interact with miR-495-3p to enhance metastatic ability of ovarian cancer cells via regulation of TPD52 [14]. A microarray-based study in ovarian cancer has shown differential expression of LINC01133 and miR-205 in ovarian cancer samples versus non-cancerous samples [15]. Contrary to the study conducted by Liu et al. [14], LINC01133 has been shown to repress proliferation, invasiveness and migration of ovarian cancer cells [15]. Functionally, LINC01133 could bind with miR-205 and subsequently regulate expression of LRRK2 [15].

Over-expression of LINC01133 in cervical cancer cells has increased their proliferation and metastatic ability while reducing their apoptosis. LINC01133 silencing has inhibited their malignant phenotype. Functionally, up-regulation of LINC01133 results in reduction of miR-30a-5p levels and enhancement of FOXD1 levels [16].

LINC01133 has also been shown to regulate malignant behavior of triple negative breast cancer cells. In fact, LINC01133 could sufficiently promote phenotypic and growth features of cancer stem cells. This lncRNA directly mediates the mesenchymal stem/stromal cells-induced miR-199a-FOXP2 axis. LINC01133 can also regulate expression of the pluripotency-determining gene KLF4 [17].

LINC01133 has also been revealed to be up-regulated in pancreatic cancer cells in association with higher DKK1 methylation and up-regulation of genes involved in the Wnt signaling pathway. LINC01133 binds with DKK1 promoter, inducing H3K27 trimethylation and decreasing its expression. However, Wnt-5a, MMP-7, and β-catenin levels have been found to be up-regulated following LINC01133 binding. Over-expression of LINC01133 has promoted proliferative potential and invasiveness of pancreatic cancer cells [18].

## Hepatocellular carcinoma

Up-regulation of LINC01133 in hepatocellular cancer cells has enhanced proliferation of these cells and induced aggressive phenotype in these cells. Mechanistically, LINC01133 sponges miR-199a-5p and increases expression of SNAI1, facilitating epithelial-mesenchymal transition (EMT) program in these cells. Moreover, LINC01133 has a functional interaction with Annexin A2 (ANXA2) to induce activity of ANXA2/STAT3 axis [19].

## Lung cancer

LINC01133 silencing has been shown to decrease proliferative ability, migratory potential and invasiveness of non-small cell lung cancer cells and induce cell cycle arrest at G1/S stage. Mechanistically, LINC01133 has interaction with EZH2 and LSD1 to recruit these proteins to the promoter regions of KLF2, P21 or E-cadherin promoters to suppress their transcription [20].

## Gastrointestinal cancers

LINC01133 has been shown to be down-regulated in gastric cancer cell lines. LINC01133 silencing has enhanced proliferation and migration, and induced the EMT program in gastric cancer cells, while its up-regulation has induced opposite impact. Based on the bioinformatics analyses and luciferase assay, miR-106a-3p has been found to be directly targeted by LINC01133. Mechanistically, miR-106a-3p can target adenomatous polyposis coli (APC) gene and decrease its expression. Taken together, LINC01133/miR-106a-3p has been found as a functional axis in suppression of EMT and metastasis through decreasing activity of the Wnt/β-catenin pathway via affecting APC levels [21]. Another study has shown that LINC01133 can up-regulate SST via binding to miR-576-5p. Up-regulation miR-576-5p or inhibition of SST has upturned the biological effects of LINC01133 in gastric cancer cells. Thus, LINC01133 up-regulation can suppress development of gastric cancer through decreasing expression of miR-576-5p and enhancing SST levels [22].

**Table 1:** Expression of LINC01133 in cell lines

Tumor/ disorder type	Interacting molecules and pathways	Cell lines	Function	References
Gastric cancer	miR-106a-3p, APC, Wnt/β-catenin pathway	SUN-216, BGC-823, AGS, BGC-803, NUGC4, MKN74, MKN45, SGC-7901, HGC-27 and GES-1	∆ LINC01133: ↑ proliferation, ↑ migration, ↑ EMT process	[21]
	miR-576-5p, SST	GC cells	↑ LINC01133: ↓ malignant phenotypes	[22]
Epithelial ovarian cancer	miR-495-3p, TPD52	SKOV3, A2780, IOSE8	↑ LINC01133: ↑ migration, ↑ invasion	[14]
Ovarian cancer	miR-205, LRRK2	IOSE80, SKOV-3, HO-8910, and OVCAR-8	↑ LINC01133: ↓ proliferation, ↓ migration, ↓ invasion	[15]
Hepatocellular carcinoma	PI3K/AKT signaling pathway	HepG2, Hep3B, MHCC-97 L, SK-Hep-1, and MHCC-97 H, HL-7702	∆ LINC01133: ↓ proliferation, ↓ migration, ↓ invasion, ↓ colony formation, ↑ apoptosis, ↑ G1 phase arrest	[23]
	miR-199a-5p, SNAI1, EMT, ANXA2/STAT3/cyclin D1 signaling	MHCC97L, MHCC97H, and HCCLM3, Hep3B, HepG2, PLC/PRF/5, and Huh7	∆ LINC01133: ↓ proliferation, ↓ migration, ↓ invasion, ↓ colony formation ↑ LINC01133: ↑ proliferation, ↑ migration, ↑ invasion, ↑ colony formation, ↑ EMT process	[19]
Cervical cancer	miR-4784, AHDC1	NC104, Hela, ME-180, C33A and MS751	∆ LINC01133: ↓ proliferation, migration, ↓ invasion, ↓ EMT process	[24]
	miR-30a-5p, FOXD1	HeLa, HT-3, C33A, SiHa	↑ LINC01133: ↑ proliferation, ↑ migration, ↑ invasion, ↓ apoptosis	[16]
Breast cancer	EZH2, SOX4	MDA-MB‐231, SKBR‐3, MDA‐MB‐468, ZR‐75‐1, BT474, MCF‐7 and T47D, MCF‐10 A	∆ LINC01133: ↑ viability, ↑ migration, ↑ invasion ↑ LINC01133: ↓ viability, ↓ migration, ↓ invasion	[25]
	miR-199a, FOXP2, KLF4 (Pluripotency Master Regulator)	DA-MB-231, MDA-MB-468, HCC1937, T47D, MCF7, ZR-75-1, BT-20, HCC1143, BT549, and Hs578T, HCC70, 4T1, and MCF10A	↑ LINC01133: ↑ SC-Like Traits in TNBC Cells	[17]
Nasopharyngeal carcinoma	YBX1	NP69, CNE-1, CNE-2, 5-8 F, 6-10B, and SUNE-1	∆ LINC01133: ↑ proliferation, ↑ migration, ↑ invasion, ↑ colony formation, ↑ EMT process ↑ LINC01133: ↓ proliferation, ↓ migration, ↓ invasion, ↓ colony formation, ↓ EMT process	[26]
Renal cell carcinoma	miR-30b-5p, Rab3D	HKC, ACHIN, A498, SN12PM6, and 786-O	∆ LINC01133: ↓ proliferation, ↓ migration, ↓ invasion	[27]
Endometrial carcinoma		Ishikawa and HEC-1-A cells	∆ LINC01133: ↓ proliferation, migration, ↓ invasion	[28]
Pancreatic cancer	C/EBPβ, CCNG1	BXPC3, CFPAC1, PANC1, and SW1990, CAPAN-2	∆ LINC01133: ↓ proliferation	[29]
	DKK1, Wnt signaling pathway	SW1990, Capan1, AsPc1, PANC − 1, BxPC − 3, and HPDE	↑ LINC01133: ↑ growth, ↑ proliferation, ↑ migration, ↑ metastasis, and ↑ invasion	[18]
	miR-216a -5p, TPT1, mTORC1 pathway	SW1990, PANC1, Capan-2, BxPC-3, and HPDE6	↑ miR-216a -5p (a target of LINC01133): ↓ proliferation, ↓ colony formation, ↑ cell cycle arrest ∆ LINC01133: ↓ proliferation, migration, ↓ invasion	[30]
	Periostin, EZH2, AXIN2, Wnt/β-catenin pathway	CFPAC-1, AsPC-1, Panc-1, SW1990, HPDE, human PSCs	∆ LINC01133: ↓ proliferation, ↓ migration, ↓ invasion, ↑ apoptosis ↑ LINC01133: ↑ proliferation, ↑ migration, ↑ invasion, ↓ apoptosis Periostin up-regulated LINC01133.	[31]
Oral squamous cell carcinoma	GDF15	NOK, CAL27, HN4, and 293FT	∆ LINC01133: did not affect proliferation, ↑ migration, ↑ invasion	[32]
Osteosarcoma	miR-422a	MG63, Saos-2, HOS, U2-OS, NHOst, and HEK-293	∆ LINC01133: ↓ proliferation, ↓ migration, ↓ invasion	[33]
Colorectal cancer	TGF-β signaling pathway, SRSF6	HT29, HCT8, LS513, SW620, and HCT11	∆ LINC01133: ↑ EMT process, ↑ metastasis TGF-β signaling pathway inhibited LINC01133.	[34]
Lung cancer	KLF2, P21 and E-cadherin, EZH2 and LSD1	PC9, SPC-A1, NCI-H1975, H1299, and A549, H520, H1703, and SK-MES-1	∆ LINC01133: ↓ proliferation, ↓ migration, ↓ invasion, ↑ apoptosis	[20]
		H1703	∆ LINC01133: ↓ migration, ↓ invasion	[35]
Bladder cancer	Wnt signaling pathway	V-HUC‐1, T24 and J82	↑ LINC01133: ↓ proliferation, ↓ migration, ↓ invasion	[36]

∆: knock-down or deletion, SC: stem cell, TNBC: triple-negative breast cancers

## Animal studies

Up-regulation of LINC01133 hepatocellular cancer cells has enhanced growth of hepatocellular carcinoma and lung metastasis in animal models, while its silencing has led to opposite effects [19]. An experiment in animal model of epithelial ovarian cancer has shown that up-regulation of this lncRNA has enhanced the metastatic ability of cells [14]. However, another study has reported enhancement of tumor weigh and volume as well as increase in metastasis following LINC01133 silencing [15].

Up-regulation of LINC01133 has reduced progression and metastasis of gastric cancer cells [21]. Similarly, experiments in an animal model of breast cancer have revealed that down-regulation of LINC01133 enhances the metastatic ability of malignant cells [25]. In order to assess the impact of LINC01133 in inhibition of colorectal cancer cells metastasis in vivo, Kong et al. have injected LINC01133-silenced HT29 cells into NOD/SCID mice. They have reported higher metastasis in the LINC01133 silenced group compared with the control group [34] (Table 2).

**Table 2:** Function of LINC01133 in animal models

Tumor/disorder type	Animal models	Results	References
Gastric cancer	Immunodeficient BABL/c female nude mice	↑ LINC01133: ↓ GC progression and ↓ metastasis	[21]
	Tumor-bearing nude mice	↑ LINC01133: ↓ tumor growth	[22]
Epithelial ovarian cancer	Female athymic BALB/c nude mice	↑ LINC01133: ↑ metastasis	[14]
Ovarian cancer	Female BALB/c nude mice	∆ LINC01133: ↑ tumor weight, ↑ tumor volume, ↑ metastasis	[15]
Hepatocellular carcinoma	Female BALB/c nude mice	∆ LINC01133: ↓ tumor weight, ↓ PI3K/AKT signaling activity	[23]
	Male BALB/c nu/nu mice	↑ LINC01133: ↑ tumor volume, ↑ metastasis	[19]
Breast cancer	Female nude mice	∆ LINC01133: ↑ metastasis	[25]
Nasopharyngeal carcinoma	Immunodeficient male BALB/c nude mice	↑ LINC01133: ↓ metastasis	[26]
Renal cell carcinoma	Female nude BALB/7 mice	∆ LINC01133: ↓ tumor weight, ↓ tumor volume	[27]
Pancreatic cancer	BALB/c nude mice	∆ LINC01133: ↓ tumor weight, ↓ proliferation	[29]
	Male BALB/c-nu nude mice	↑ LINC01133: ↑ tumor weight, ↑ metastasis ∆ LINC01133: ↓ tumor weight, ↓ metastasis	[18]
	SCID mice	↑ miR-216a -5p (a target of LINC01133): ↓ tumor weight, ↓ metastasis	[30]
	Male immunodeficient BALB/c nude mice	∆ LINC01133: ↓ tumor growth, ↓ tumor weight and ↓ tumor volume	[31]
Colorectal cancer	Male NOD–SCID–gamma mice	∆ LINC01133: ↑ metastasis	[34]
Lung cancer	Female athymic BALB/c nude mice	∆ LINC01133: ↓ tumor weight, ↓ tumor growth	[20]
Bladder cancer	Male BALB/c-nu mice	↑ LINC01133: ↓ tumor growth	[36]

∆: knock-down or deletion, GC: Gastric cancer, SCID: severe combined immunodeficient

## Human studies

Expression of LINC01133 has been shown to be down-regulated in clinical samples obtained from gastric cancer patients in correlation with progression of gastric cancer and metastasis [21]. Similar results have been obtained from expression assays in nasopharyngeal cancer [26], oral [32]/esophageal squamous cell carcinoma [37] and colorectal cancer [38] (Table 3).

Through analysis of whole genome sequencing data of hepatocellular cancer samples and matched noncancerous specimens, Yin et al. have reported increased in genomic copy numbers of LINC01133 in cancerous samples in correlation with up-regulation of LINC01133 and poor prognosis of affected individuals [19]. Similarly, assessment of expression profile of cervical cancer samples in TCGA database has revealed up-regulation of LINC01133 levels in these samples [24]. Another study has confirmed up-regulation of LINC01133 in cervical cancer samples and reported association between its levels and advanced T stage and negative HPV infection [16]. Besides, LINC01133 has been found to be up-regulated in pancreatic cancer and osteosarcoma. Dysregulation of LINC01133 in clinical samples has been frequently associated with malignant features and poor patients’ outcome. However, different experiments in in ovarian, breast and lung cancers have reported conflicting results regarding the pattern of expression of LINC01133 in cancerous versus non-cancerous samples (Table 3).

**Table 3:** Dysregulation of LINC01133 in clinical samples

Tumor/disorder type	Samples	Expression (tumor versus non-tumoral samples)	Kaplan–Meier analysis (effect of LINC01133 up-regulation)	Univariate/multivariate cox regression	Association of LINC01133 expression with Clinical/ pathological factors	References
Gastric cancer	200 pairs of tumor tissues and AdNTs	Down	Longer 5-year OS and 5-year PFS	LINC01133 was found to be an independent protective predictor of OS and PFS.	Low expression levels of LINC01133 were correlated with greater size of tumor, advanced T stage, lymphatic invasion, advanced TNM stage, and infiltration of peritumoral tissues.	[21]
	GEO database (GSE70880, GSE51308, GSE84787, GSE50710, GSE79973, GSE19826, GSE54129) plus 50 pairs of tumor tissues and AdNTs	Down			Gender (higher in females than males)	[39]
Epithelial ovarian cancer	25 EOC tissues and 4 normal ovarian surface epithelial tissue	Up	Lower OS			[14]
Ovarian cancer	GEO database (GSE14407, GSE38666, and GSE83693) plus 50 ovarian cancer tissues and 30 normal ovarian tissues	Down	Longer OS			[15]
Hepatocellular carcinoma	667 patients with primary HCC (three different cohorts)	Up	CNV in LINC01133 was associated with lower OS.	The CNV of LINC01133 was an independent prognostic factor for patient survival.		[19]
Cervical cancer	TCGA database	Up				[24]
	50 pairs of tumor tissues and AdNTs, TCGA database from GEPIA	Up			Higher T stage and negative HPV infection	[16]
	TCGA database	Up				[40]
	115 CESC cases, 79 cases of CIN and 101 healthy controls	Up in CESC and CIN				[41]
Breast cancer	74 pairs of tumor tissues and AdNTs	Down	Longer OS	Low expression levels of LINC01133, and lymph node metastasis and TNM stage was found to be independent prognostic indicators for patients.	Down-regulation levels are associated with lymph node metastasis and advanced TNM stage.	[25]
	TCGA database (derived from TANRIC database) GEO database (GSE76275, GSE76124, GSE36771, and GSE3744)	Up	Poor OS			[17]
	Lnc2Cancer database 79 pairs of luminal A and B BC tissues and AdNTs	Down in luminal A and B BC tissues				[42]
Nasopharyngeal carcinoma	15 NPC tissues and 6 normal nasopharyngeal epithelium tissues GEO database (GSE12452: 31 NPC and 10 normal nasopharyngeal samples)	Down				[26]
Renal cell carcinoma	34 pairs of tumor tissues and AdNTs	Up				[27]
Pancreatic cancer	132 patients with PDAC GEO database: (GSE15471, GSE16515, and GSE32676) and TCGA database	Up	Poor OS and DFS		Tumor size, T stage, TNM stage, histological grade, disease-free status, and mutation count	[29]
	GEO database: (GSE15471 and GSE16515: 75 PDAC tissue samples and 55 normal pancreatic)	Up	Shorter OS			[43]
	GSE32676 and GSE16515	Up				[18]
	40 pairs of tumor tissues and AdNTs	Down-regulation of miR-216a -5p (a target of LINC01133)			Down-regulation levels of miR-216a -5p are associated with peripancreatic lymphatic metastasis, perineural invasion and advanced TNM stage.	[30]
	32 pairs of tumor tissues and AdNTs 80 pairs of tumor tissues and AdNTs	Up	Poor OS		Higher TNM stage	[31]
Oral squamous cell carcinoma	50 pairs of tumor tissues and AdNTs	Down	Longer OS			[32]
Esophageal squamous cell carcinoma	149 pairs of tumor tissues and AdNTs	Down	Poorer OS and PFS	LINC01133 was found to be an independent favorable predictor of OS and PFS. (LINC01133 expression Combination, TNM stage and drinking status, showed to be the best predictive value in patients.)	Low levels of LINC01133 were associated with ever smoking, ever drinking, large tumor size, greater depth of tumor invasion, lymph node metastasis, and advanced TNM stage.	[37]
Osteosarcoma	27 pairs of tumor tissues and AdNTs	Up	Lower OS and poorer prognosis			[33]
Colorectal cancer	187 pairs of tumor tissues and AdNTs	Down	Longer OS	LINC01133 was found to be an independent prognostic factor.	Low levels of LINC01133 were associated with lymph node metastasis, distant metastasis, N classification, and TNM stage.	[38]
	219 pairs of tumor tissues and AdNTs GEO database: (GSE40967)	Down	Longer OS longer OS and RFS		Low levels of LINC01133 were associated with distant metastasis.	[34]
Lung cancer	GEO database: (GSE18842 and GSE19804) 68 pairs of tumor tissues and AdNTs	Up	Lower OS and poorer prognosis		Tumor size, advanced pathological stage, and lymph node metastasis	[20]
	GEO database: (GSE10245)	Up in LSCC but not in LAD samples	Lower OS			[35]

AdNTs, adjacent non-cancerous tissues; OS, overall survival; PFS, progression-free survival; EOC, epithelial ovarian cancer; CNV, copy number variation; CESC, cervical squamous carcinoma; CIN, cervical intraepithelial neoplasia; BC, breast cancer; NPC, Nasopharyngeal carcinoma; DFS, disease-free survival; RFS, recurrent free survival; LAD, Lung adenocarcinoma; LSCC, lung squamous cell cancer

## Discussion

LINC01133 is an important lncRNA in the process of carcinogenesis. However, it can exert dissimilar roles in this process. In gastric cancer [21], nasopharyngeal cancer [26], oral [32]/esophageal squamous cell carcinoma [37] and colorectal cancer [38], it has a tumor suppressor role. On the other hand, in hepatocellular carcinoma [19], cervical cancer [16], pancreatic cancer [29] and osteosarcoma [33], LINC01133 has been demonstrated to exert oncogenic effects. Finally, in ovarian [14, 15] and breast [17, 25] data is conflicting about the role of this lncRNA. Animal studies have also revealed conflicting results regarding the oncogenic versus tumor suppressor role of LINC01133 in different tissues.

Interaction between LINC01133 and miRNAs is a well-appreciated way of contribution of this lncRNA in the carcinogenesis. miR-106a-3p, miR-576-5p, miR-495-3p, miR-205, miR-199a-5p, miR-4784, miR-30a-5p, miR-199a, miR-30b-5p, miR-216a -5p and miR-422a are the main miRNAs mediating the effects of LINC01133 in this process (reviewed in Table 1). PI3K/AKT [23], STAT3 [19], Wnt [18], mTORC1 [30] and TGF-β [34] signaling pathways have also been shown to be affected by LINC01133. Notably, LINC01133 can affect EMT process in liver, gastric, colorectal, cervical and nasopharyngeal cancers. Thus, dysregulation of this lncRNA can enhance metastatic ability of cancer cells.

LINC01133 levels have been used to predict prognosis of cancer in different tissues (reviewed in Table 3). Dysregulation of LINC01133 has been found to affect clinical outcomes in different studies. However, since it can exert dissimilar roles in different tissues, the impact of LINC01133 down-/up-regulation on clinical outcome depends on the tissue origin.

Data about the mechanisms of dysregulation of LINC01133 in cancer is scarce. However, the presence of CNVs has been shown to affect its expression [19]. Moreover, there is no clear elucidation for tissue-specific effects of this lncRNA in the carcinogenesis. Based on the presence of conflicting results about the role of LINC01133 in the evolution of cancer, therapeutic targeting of this lncRNA should be considered with caution. Moreover, it is necessary to design novel methods for specific delivery of LINC01133-targeting therapeutic modalities to target tissues.

## Data Availability

The analyzed data sets generated during the study are available from the corresponding author on reasonable request.
